# Supplementary material for: No Compelling Evidence that Preferences for Facial Masculinity Track Changes in Women’s Hormonal Status
Source: Psychol Sci. 2018 Apr 30;29(6):996–1005. doi: 10.1177/0956797618760197 (PMC6099988; doi:10.1177/0956797618760197)
Supplement: JonesSupplementalMaterial – Supplemental material for No Compelling Evidence That Preferences for Facial Masculinity Track Changes in Women’s Hormonal Status [file JonesSupplementalMaterial.pdf]

Male Facial Masculinity

BC Jones et al. (ben.jones@glasgow.ac.uk (mailto:ben.jones@glasgow.ac.uk))

- Overview
- Hypothesis 1
  - Descriptive stats: data_hormones
    - Preferences
    - Hormones
  - Analyses H1: Hormones
    - E + P + E*P:
    - E + P + EPratio:
    - T + C:
    - T + C + T*C:
  - Analyses H1s: Hormones (+ session order)
    - E + P + E*P: (+ session order)
    - E + P + EPratio: (+ session order)
    - T + C: (+ session order)
    - T + C + T*C: (+ session order)
  - Descriptive stats: data_hormones_partner
  - Analyses H1p: Hormones (+ partnership status)
    - E + P + E*P: (+ partnership status)
      - E + P + E*P: (single women only to interpret interaction)
      - E + P + E*P: (partnered women only to interpret interaction)
    - E + P + EPratio: (+ partnership status)
    - T + C: (+ partnership status)
    - T + C + T*C: (+ partnership status)
  - Analyses H1ps: Hormones (+ session order, + partnership status)
    - E + P + E*P: (+ session order, + partnership status)
      - E + P + E*P: (single women only to interpret interaction)
      - E + P + E*P: (partnered women only to interpret interaction)
    - E + P + EPratio: (+ session order, + partnership status)
    - T + C: (+ session order, + partnership status)
    - T + C + T*C: (+ session order, + partnership status)
- Hypothesis 2
  - Descriptive stats: data_between
  - Analyses H2: Pill
  - Analyses H2p: Pill (+ partnership status)
    - Pill (single women only to interpret interaction)
    - Pill (partnered women only to interpret interaction)
- Hypothesis 3
  - Descriptive stats: data_pillbreak
  - Analyses H3: Pill-break
  - Analyses H3p: Pill-break (+ partnership status)
    - Pill break (single women only to interpret interaction)
    - Pill break (partnered women only to interpret interaction)
- Hypothesis 4

- Descriptive stats: data_pill_switchers
  - Interval between pill use and non-use testing blocks
- Analyses H4: Pill-switch
- Analyses H4p: Pill-switch (+ partnership status change)

Overview

This supplemental information contains the R code for data analysis of **male facial masculinity** preferences reported in the manuscript (data are publicly available at https://osf.io/9b4y7/ (https://osf.io/9b4y7/)).

Hypothesis 1. Do preferences track changes in measured steroid hormone levels in women not using hormonal contraceptives?

Women reporting no use of hormonal contraceptives and for whom at least two test sessions with valid hormone levels are available.

Hypothesis 2. Do women not using hormonal contraceptives show stronger preferences than women using the combined oral contraceptive pill?

Women reporting use of the combined oral contraceptive pill or no use of hormonal contraceptives across all test sessions.

Hypothesis 3. Do preferences of women using the combined oral contraceptive pill change when they are taking inactive pills?

Women who were tested during a scheduled break from use of the combined oral contraceptive pill.

Hypothesis 4. Do preferences change when women start or stop using the combined oral contraceptive pill?

Women who switched from using no hormonal contraceptive to using the combined oral contraceptive pill (or vice versa) between blocks of test sessions.

```
## Loading tidyverse: ggplot2
## Loading tidyverse: tibble
## Loading tidyverse: tidyr
## Loading tidyverse: readr
## Loading tidyverse: purrr
## Loading tidyverse: dplyr
```

```
## Conflicts with tidy packages ----------------------------------------------
```

```
## filter(): dplyr, stats
## lag():    dplyr, stats
```

```
## Loading required package: Matrix
```

```
##
## Attaching package: 'Matrix'
```

```
## The following object is masked from 'package:tidyr':
##
##     expand
```

```
##
## Attaching package: 'lmerTest'
```

```
## The following object is masked from 'package:lme4':
##
##     lmer
```

```
## The following object is masked from 'package:stats':
##
##     step
```

```
##
## Attaching package: 'lubridate'
```

```
## The following object is masked from 'package:base':
##
##     date
```

```
# load data frames from Jones_hormones_data.Rmd
data_hormones <- readRDS("data_hormones.Rda")
data_hormones_partner <- readRDS("data_hormones_partner.Rda")
data_between <- readRDS("data_between.Rda")
data_between_partner <- readRDS("data_between_partner.Rda")
data_pillbreak <- readRDS("data_pillbreak.Rda")
data_pillbreak_partner <- readRDS("data_pillbreak_partner.Rda")
data_pill_switchers <- readRDS("data_pill_switchers.Rda")
data_pill_switchers_partner <- readRDS("data_pill_switchers_partner.Rda")
```

```
# filter only sexual dimorphism manipulations for male faces in the analyses b
elow
theManip <- "sexdim"
theFaceSex <- "men"
confint_method <- "Wald" # c("profile", "Wald", "boot")
```

```
# calculate standard errors
se <- function(x, na.rm = FALSE) {
  if (na.rm) {
    the.SE <- sqrt(var(x,na.rm=TRUE)/length(na.omit(x)))
  } else {
    the.SE <- sqrt(var(x,na.rm=FALSE)/length(x))
  }

  return(the.SE)
}

# short summaries for lmerTest
mySummary <- function(lmer_summary) {

  coefTable <- lmer_summary$coefficients %>%
    round(3) %>%
    as.data.frame() %>%
    rownames_to_column()

  if (ncol(coefTable)>5) {
    coefTable <- coefTable %>%
      mutate(
        sig = ifelse(.[6]<.001, "***",
              ifelse(.[6]<.01,   "**",
              ifelse(.[6]<.05,   "*",
              ifelse(.[6]<.10,   "+", "")))))
  }

  return(list(lmer_summary$ngrps, kable(coefTable)))
}
```

Hypothesis 1

Do preferences track changes in measured steroid hormone levels in women not using hormonal contraceptives?

For tests of effects of endogenous hormones. Women reporting no use of hormonal contraceptives and for whom at least two test sessions with valid hormone levels are available (all relationship statuses).

Descriptive stats: data_hormones

Preferences

```
# create mean DV for all ratings by oc_id
stats_overall <- filter(data_hormones, face_sex==theFaceSex, manip==theManip)
%>%
  group_by(oc_id) %>%
  summarise(
    overall_rating.c = mean(rating.c)
  ) %>%
  ungroup() %>%
  group_by() %>%
  summarise(
    context="overall",
    n = n_distinct(oc_id),
    mean_dv = mean(overall_rating.c),
    sd_dv = sd(overall_rating.c),
    se_dv = se(overall_rating.c)
  )

# create mean DV splitting by context
stats_context <- filter(data_hormones, face_sex==theFaceSex, manip==theManip)
%>%
  group_by(oc_id, context) %>%
  summarise(
    context_rating.c = mean(rating.c)
  ) %>%
  group_by(context) %>%
  summarise(
    n = n_distinct(oc_id),
    mean_dv = mean(context_rating.c),
    sd_dv = sd(context_rating.c),
    se_dv = se(context_rating.c)
  )

rbind(stats_overall, stats_context)
```

```
## # A tibble: 3 x 5
##   context     n   mean_dv     sd_dv      se_dv
## *   <chr> <int>     <dbl>     <dbl>      <dbl>
## 1 overall   351 0.3461916 0.4794891 0.02559323
## 2      LT   351 0.3214278 0.5017172 0.02677968
## 3      ST   351 0.3708437 0.5076745 0.02709766
```

Hormones

```
filter(data_hormones, face_sex==theFaceSex, manip==theManip) %>%
    group_by() %>%
    summarise(
      mean_prog = mean(prog, na.rm = TRUE),
      sd_prog =sd(prog, na.rm = TRUE),
      se_prog =se(prog, na.rm = TRUE),
      mean_estr = mean(estr, na.rm = TRUE),
      sd_estr =sd(estr, na.rm = TRUE),
      se_estr =se(estr, na.rm = TRUE),
      mean_test = mean(test, na.rm = TRUE),
      sd_test =sd(test, na.rm = TRUE),
      se_test =se(test, na.rm = TRUE),
      mean_cort = mean(cort, na.rm = TRUE),
      sd_cort =sd(cort, na.rm = TRUE),
      se_cort =se(cort, na.rm = TRUE)
  ) %>% gather("stat", "value", 1:length(.)) %>%
    mutate(value = round(value, 4)) %>%
    separate(stat, c("stat", "hormone")) %>%
    spread(stat, value)
```

```
## # A tibble: 4 x 4
##   hormone     mean      sd     se
## *   <chr>    <dbl>   <dbl>  <dbl>
## 1    cort   0.2288  0.1645 0.0025
## 2    estr   3.2945  1.2718 0.0195
## 3    prog 148.5481 96.1287 1.4812
## 4    test  87.6616 27.1943 0.4172
```

Analyses H1: Hormones

E + P + E*P:

Testing for effects of estradiol, progesterone, and their interaction on preferences

```
model_h1_EP <- lmer(rating.c ~ estr.s * prog.s * context.e +
                          (1 | oc_id) +
                          (0 + estr.s:prog.s:context.e || oc_id) +
                          (1 | block:oc_id) +
                          (0 + estr.s:prog.s:context.e|| block:oc_id) +
                          (1 | block:oc_id:date) +
                          (0 + context.e || block:oc_id:date),
                     data = filter(data_hormones,
                                   face_sex==theFaceSex,
                                   manip==theManip),
                     REML = FALSE)
summary_h1_EP <- summary(model_h1_EP)
mySummary(summary_h1_EP)
```

```
## [[1]]
## block:oc_id:date      block:oc_id           oc_id
##             2092              444             351
##
## [[2]]
##
##
## rowname                    Estimate   Std. Error         df   t value   Pr(
>|t|)  sig
## ------------------------  ---------  -----------  ---------  --------  ----
-----  ----
## (Intercept)                   0.347        0.026    362.110    13.467
0.000  ***
## estr.s                       -0.005        0.051   1733.893    -0.104
0.917
## prog.s                       -0.010        0.043   1735.838    -0.221
0.825
## context.e                     0.052        0.013   2081.180     4.095
0.000  ***
## estr.s:prog.s                -0.099        0.253   1784.753    -0.393
0.695
## estr.s:context.e              0.047        0.082   1723.701     0.569
0.569
## prog.s:context.e              0.025        0.073   1560.698     0.344
0.731
## estr.s:prog.s:context.e       0.362        0.412     72.798     0.879
0.382
```

```
confint(model_h1_EP, method = confint_method) %>% as.data.frame() %>% rownames
_to_column() %>% filter(!is.na(`2.5 %`))
```

```
##                   rowname       2.5 %     97.5 %
## 1             (Intercept)  0.29686369 0.39799451
## 2                  estr.s -0.10517476 0.09454539
## 3                  prog.s -0.09478402 0.07553965
## 4               context.e  0.02718444 0.07710258
## 5           estr.s:prog.s -0.59503289 0.39635879
## 6        estr.s:context.e -0.11356992 0.20657516
## 7        prog.s:context.e -0.11742755 0.16748868
## 8 estr.s:prog.s:context.e -0.44529767 1.16952870
```

E + P + EPratio:

Testing for effects of estradiol, progesterone, and their ratio on preferences

```
model_h1_EP_ep_ratio <- lmer(rating.c ~ estr.s * context.e +
                                        prog.s * context.e +
                                        ep_ratio.s * context.e +
                          (1 | oc_id) +
                          (0 + estr.s:context.e + prog.s:context.e + ep_ratio.
s:context.e || oc_id) +
                          (1 | block:oc_id) +
                          (0 + estr.s:context.e + prog.s:context.e + ep_ratio.
s:context.e|| block:oc_id) +
                          (1 | block:oc_id:date) +
                          (0 + context.e || block:oc_id:date),
                     data = filter(data_hormones,
                                   face_sex==theFaceSex,
                                   manip==theManip),
                     REML = FALSE)
summary_h1_EP_ep_ratio <- summary(model_h1_EP_ep_ratio)
mySummary(summary_h1_EP_ep_ratio)
```

```
## [[1]]
## block:oc_id:date      block:oc_id            oc_id
##             2092              444              351
##
## [[2]]
##
##
## rowname                   Estimate   Std. Error         df   t value   Pr(>|t
|)  sig
## ---------------------  ---------  -----------  ---------  --------  -------
--  ----
## (Intercept)                0.347        0.026    356.819    13.482      0.0
00  ***
## estr.s                     0.002        0.052   1740.901     0.031      0.9
75
## context.e                  0.056        0.012   2087.545     4.567      0.0
00  ***
## prog.s                    -0.029        0.049   1721.956    -0.594      0.5
52
## ep_ratio.s                -0.018        0.025   1691.625    -0.717      0.4
73
## estr.s:context.e           0.054        0.084   2092.809     0.647      0.5
18
## context.e:prog.s           0.026        0.083   2090.185     0.317      0.7
51
## context.e:ep_ratio.s      -0.006        0.043   2084.905    -0.132      0.8
95
```

```
confint(model_h1_EP_ep_ratio, method = confint_method) %>% as.data.frame() %>%
rownames_to_column() %>% filter(!is.na(`2.5 %`))
```

```
##               rowname       2.5 %     97.5 %
## 1          (Intercept)  0.29619719 0.39696665
## 2               estr.s -0.10019500 0.10345275
## 3            context.e  0.03190113 0.07986220
## 4               prog.s -0.12594936 0.06734871
## 5           ep_ratio.s -0.06779680 0.03147571
## 6     estr.s:context.e -0.10968230 0.21781394
## 7     context.e:prog.s -0.13601061 0.18844409
## 8 context.e:ep_ratio.s -0.08969433 0.07841501
```

T + C:

Testing for effects of testosterone and coritsol on preferences

```
# Converges when we model random slopes for individual predictors and the inte
raction on oc_id
model_h1_TC <- lmer(rating.c ~ test.s * context.e + cort.s * context.e +
                          (1 | oc_id) +
                          (0 + test.s*context.e + cort.s*context.e || oc_id) +
                          (1 | block:oc_id) +
                          (0 + test.s*context.e + cort.s*context.e|| block:oc_
id) +
                          (1 | block:oc_id:date) +
                          (0 + context.e || block:oc_id:date),
                     data = filter(data_hormones,
                                   face_sex==theFaceSex,
                                   manip==theManip),
                     REML = FALSE)
summary_h1_TC <- summary(model_h1_TC)
mySummary(summary_h1_TC)
```

```
## [[1]]
## block:oc_id:date      block:oc_id           oc_id
##             2117              443             350
##
## [[2]]
##
##
## rowname             Estimate   Std. Error         df   t value   Pr(>|t|)
sig
## -----------------  ---------  -----------  ---------  --------  ---------
----
## (Intercept)            0.348        0.026    351.997    13.558      0.000
***
## test.s                -0.013        0.051   1517.989    -0.261      0.794
## context.e              0.054        0.016    342.392     3.302      0.001
**
## cort.s                 0.042        0.043     77.255     0.987      0.327
## test.s:context.e      -0.036        0.077   1799.430    -0.472      0.637
## context.e:cort.s      -0.003        0.057   1780.370    -0.055      0.956
```

```
confint(model_h1_TC, method = confint_method) %>% as.data.frame() %>% rownames
_to_column() %>% filter(!is.na(`2.5 %`))
```

```
##              rowname       2.5 %     97.5 %
## 1        (Intercept)  0.29755599 0.39812117
## 2             test.s -0.11289060 0.08638938
## 3          context.e  0.02209064 0.08660596
## 4             cort.s -0.04153990 0.12583238
## 5 test.s:context.e -0.18602187 0.11387628
## 6 context.e:cort.s -0.11546374 0.10919083
```

T + C + T*C:

Testing for effects of testosterone and coritsol plus their interaction on preferences

```
# Converges when we model random slopes for individual predictors and the inte
raction on oc_id
model_h1_TCi <- lmer(rating.c ~ test.s * context.e * cort.s +
                          (1 | oc_id) +
                          (0 + test.s*cort.s*context.e || oc_id) +
                          (1 | block:oc_id) +
                          (0 + test.s*cort.s*context.e|| block:oc_id) +
                          (1 | block:oc_id:date) +
                          (0 + context.e || block:oc_id:date),
                     data = filter(data_hormones,
                                   face_sex==theFaceSex,
                                   manip==theManip),
                     REML = FALSE)
summary_h1_TCi <- summary(model_h1_TCi)
mySummary(summary_h1_TCi)
```

```
## [[1]]
## block:oc_id:date      block:oc_id           oc_id
##             2117              443              350
##
## [[2]]
##
##
## rowname                     Estimate   Std. Error          df   t value   Pr(
>|t|)  sig
## ------------------------  ---------  -----------  ---------  --------  ----
-----  ----
## (Intercept)                   0.340        0.026    363.861    13.180
0.000  ***
## test.s                       -0.008        0.051   1466.856    -0.166
0.868
## context.e                     0.056        0.017    391.077     3.257
0.001  **
## cort.s                        0.017        0.043     65.367     0.399
0.692
## test.s:context.e             -0.036        0.077   1762.675    -0.476
0.634
## test.s:cort.s                 0.510        0.213    173.893     2.393
0.018  *
## context.e:cort.s              0.003        0.059   1383.311     0.055
0.956
## test.s:context.e:cort.s      -0.081        0.290    118.611    -0.277
0.782
```

```
confint(model_h1_TCi, method = confint_method) %>% as.data.frame() %>% rowname
s_to_column() %>% filter(!is.na(`2.5 %`))
```

```
##                   rowname       2.5 %     97.5 %
## 1             (Intercept)  0.28958949 0.39076303
## 2                  test.s -0.10808618 0.09122954
## 3               context.e  0.02211887 0.08896728
## 4                  cort.s -0.06644062 0.10035490
## 5        test.s:context.e -0.18660194 0.11367372
## 6           test.s:cort.s  0.09240440 0.92831933
## 7        context.e:cort.s -0.11299765 0.11951841
## 8 test.s:context.e:cort.s -0.64980293 0.48869086
```

Analyses H1s: Hormones (+ session order)

E + P + E*P: (+ session order)

Testing for effects of estradiol, progesterone, and their interaction on preferences

```
model_h1_EP_s <- lmer(rating.c ~ estr.s * prog.s * context.e + session_n +
                          (1 | oc_id) +
                          (0 + estr.s:prog.s:context.e || oc_id) +
                          (1 | block:oc_id) +
                          (0 + estr.s:prog.s:context.e|| block:oc_id) +
                          (1 | block:oc_id:date) +
                          (0 + context.e || block:oc_id:date),
                     data = filter(data_hormones,
                                   face_sex==theFaceSex,
                                   manip==theManip),
                     REML = FALSE)
summary_h1_EP_s <- summary(model_h1_EP_s)
mySummary(summary_h1_EP_s)
```

```
## [[1]]
## block:oc_id:date      block:oc_id            oc_id
##             2092              444              351
##
## [[2]]
##
##
## rowname                    Estimate   Std. Error         df   t value   Pr(
>|t|)  sig
## ------------------------  ---------  -----------  ---------  --------  ----
-----  ----
## (Intercept)                   0.403        0.028    499.963    14.287
0.000  ***
## estr.s                       -0.025        0.051   1749.410    -0.502
0.616
## prog.s                        0.007        0.043   1726.724     0.160
0.873
## context.e                     0.052        0.013   2081.200     4.093
0.000  ***
## session_n                    -0.020        0.004    888.856    -4.924
0.000  ***
## estr.s:prog.s                -0.110        0.251   1780.634    -0.440
0.660
## estr.s:context.e              0.046        0.082   1731.563     0.567
0.571
## prog.s:context.e              0.025        0.073   1564.314     0.346
0.730
## estr.s:prog.s:context.e       0.357        0.413     73.123     0.866
0.389
```

```
confint(model_h1_EP_s, method = confint_method) %>% as.data.frame() %>% rownam
es_to_column() %>% filter(!is.na(`2.5 %`))
```

```
##                     rowname       2.5 %      97.5 %
## 1              (Intercept)  0.34790582  0.45853369
## 2                   estr.s -0.12495796  0.07401377
## 3                   prog.s -0.07784856  0.09169048
## 4                context.e  0.02716474  0.07708351
## 5                session_n -0.02823991 -0.01215962
## 6            estr.s:prog.s -0.60234695  0.38151110
## 7         estr.s:context.e -0.11380008  0.20635152
## 8         prog.s:context.e -0.11735560  0.16759816
## 9 estr.s:prog.s:context.e -0.45159613  1.16612625
```

E + P + EPratio: (+ session order)

Testing for effects of estradiol, progesterone, and their ratio on preferences

```
model_h1_EP_ep_ratio_s <- lmer(rating.c ~ estr.s * context.e +
                                          prog.s * context.e +
                                          ep_ratio.s * context.e + session_n +
                          (1 | oc_id) +
                          (0 + estr.s:context.e + prog.s:context.e + ep_ratio.
s:context.e || oc_id) +
                          (1 | block:oc_id) +
                          (0 + estr.s:context.e + prog.s:context.e + ep_ratio.
s:context.e|| block:oc_id) +
                          (1 | block:oc_id:date) +
                          (0 + context.e || block:oc_id:date),
                     data = filter(data_hormones,
                                   face_sex==theFaceSex,
                                   manip==theManip),
                     REML = FALSE)
summary_h1_EP_ep_ratio_s <- summary(model_h1_EP_ep_ratio_s)
mySummary(summary_h1_EP_ep_ratio_s)
```

```
## [[1]]
## block:oc_id:date      block:oc_id           oc_id
##             2092              444             351
##
## [[2]]
##
##
## rowname                  Estimate   Std. Error          df    t value   Pr(>|t
|)  sig
## ---------------------  ---------  -----------  ----------  ---------  -------
--  ----
## (Intercept)                 0.402        0.028     493.868     14.302      0.0
00  ***
## estr.s                     -0.018        0.052    1752.360     -0.347      0.7
29
## context.e                   0.056        0.012    2087.488      4.563      0.0
00  ***
## prog.s                     -0.015        0.049    1714.073     -0.298      0.7
66
## ep_ratio.s                 -0.020        0.025    1687.992     -0.791      0.4
29
## session_n                  -0.020        0.004     888.515     -4.930      0.0
00  ***
## estr.s:context.e            0.054        0.084    2092.773      0.645      0.5
19
## context.e:prog.s            0.026        0.083    2090.138      0.316      0.7
52
## context.e:ep_ratio.s       -0.006        0.043    2084.836     -0.131      0.8
95
```

```
confint(model_h1_EP_ep_ratio_s, method = confint_method) %>% as.data.frame() %
>% rownames_to_column() %>% filter(!is.na(`2.5 %`))
```

```
##               rowname       2.5 %      97.5 %
## 1          (Intercept)  0.34719714  0.45746899
## 2               estr.s -0.11933383  0.08347172
## 3            context.e  0.03184974  0.07981145
## 4               prog.s -0.11069007  0.08146607
## 5           ep_ratio.s -0.06911229  0.02937832
## 6            session_n -0.02825967 -0.01218148
## 7     estr.s:context.e -0.10983204  0.21766519
## 8     context.e:prog.s -0.13608465  0.18837273
## 9 context.e:ep_ratio.s -0.08968998  0.07842241
```

T + C: (+ session order)

Testing for effects of testosterone and coritsol on preferences

```
# Converges when we model random slopes for individual predictors and the inte
raction on oc_id
model_h1_TC_s <- lmer(rating.c ~ test.s * context.e + cort.s * context.e + ses
sion_n +
                          (1 | oc_id) +
                          (0 + test.s*context.e + cort.s*context.e || oc_id) +
                          (1 | block:oc_id) +
                          (0 + test.s*context.e + cort.s*context.e|| block:oc_
id) +
                          (1 | block:oc_id:date) +
                          (0 + context.e || block:oc_id:date),
                     data = filter(data_hormones,
                                   face_sex==theFaceSex,
                                   manip==theManip),
                     REML = FALSE)
summary_h1_TC_s <- summary(model_h1_TC_s)
mySummary(summary_h1_TC_s)
```

```
## [[1]]
## block:oc_id:date      block:oc_id            oc_id
##             2117              443              350
##
## [[2]]
##
##
## rowname               Estimate   Std. Error         df   t value   Pr(>|t|)
sig
## -----------------  ---------  -----------  ---------  --------  ---------
----
## (Intercept)            0.402        0.028    484.840    14.351      0.000
***
## test.s                -0.014        0.051   1578.123    -0.278      0.781
## context.e              0.054        0.016    342.404     3.299      0.001
**
## cort.s                 0.047        0.043     85.025     1.099      0.275
## session_n             -0.020        0.004    777.129    -4.849      0.000
***
## test.s:context.e      -0.036        0.077   1799.454    -0.472      0.637
## context.e:cort.s      -0.003        0.057   1780.335    -0.054      0.957
```

```
confint(model_h1_TC_s, method = confint_method) %>% as.data.frame() %>% rownam
es_to_column() %>% filter(!is.na(`2.5 %`))
```

```
##            rowname       2.5 %      97.5 %
## 1      (Intercept)  0.34678595  0.45649347
## 2           test.s -0.11314404  0.08501393
## 3        context.e  0.02204139  0.08656692
## 4           cort.s -0.03701695  0.13144677
## 5        session_n -0.02738266 -0.01161808
## 6 test.s:context.e -0.18604885  0.11384184
## 7 context.e:cort.s -0.11540624  0.10924340
```

T + C + T*C: (+ session order)

Testing for effects of testosterone and coritsol plus their interaction on preferences

```
# Converges when we model random slopes for individual predictors and the inte
raction on oc_id
model_h1_TCi_s <- lmer(rating.c ~ test.s * context.e * cort.s  + session_n +
                          (1 | oc_id) +
                          (0 + test.s*cort.s*context.e || oc_id) +
                          (1 | block:oc_id) +
                          (0 + test.s*cort.s*context.e|| block:oc_id) +
                          (1 | block:oc_id:date) +
                          (0 + context.e || block:oc_id:date),
                     data = filter(data_hormones,
                                   face_sex==theFaceSex,
                                   manip==theManip),
                     REML = FALSE)
summary_h1_TCi_s <- summary(model_h1_TCi_s)
mySummary(summary_h1_TCi_s)
```

```
## [[1]]
## block:oc_id:date      block:oc_id           oc_id
##             2117              443             350
##
## [[2]]
##
##
## rowname                     Estimate   Std. Error          df   t value   Pr(
>|t|)  sig
## -----------------------  ---------  -----------  ---------  --------  ----
-----  ----
## (Intercept)                  0.394        0.028    496.821    13.988
0.000  ***
## test.s                      -0.010        0.051   1466.836    -0.196
0.845
## context.e                    0.055        0.017    391.063     3.254
0.001  **
## cort.s                       0.023        0.043     61.456     0.540
0.591
## session_n                   -0.019        0.004    787.814    -4.812
0.000  ***
## test.s:context.e            -0.036        0.077   1762.260    -0.476
0.634
## test.s:cort.s                0.497        0.212    170.717     2.338
0.021  *
## context.e:cort.s             0.003        0.059   1379.452     0.056
0.955
## test.s:context.e:cort.s     -0.081        0.290    117.349    -0.277
0.782
```

```
confint(model_h1_TCi_s, method = confint_method) %>% as.data.frame() %>% rowna
mes_to_column() %>% filter(!is.na(`2.5 %`))
```

```
##                   rowname       2.5 %      97.5 %
## 1             (Intercept)  0.33864422  0.44900805
## 2                  test.s -0.10897840  0.08918440
## 3               context.e  0.02207067  0.08892861
## 4                  cort.s -0.06051673  0.10660158
## 5               session_n -0.02724470 -0.01147466
## 6        test.s:context.e -0.18663173  0.11363595
## 7           test.s:cort.s  0.08031600  0.91303187
## 8        context.e:cort.s -0.11294262  0.11956736
## 9 test.s:context.e:cort.s -0.64976485  0.48869776
```

Descriptive stats: data_hormones_partner

```
data_hormones_partner %>%
  filter(face_sex==theFaceSex, manip==theManip) %>%
  group_by(oc_id, partner.e) %>%
  summarise(overall_rating.c = mean(rating.c)) %>%
  group_by(partner.e) %>%
  summarise(
    n= n_distinct(oc_id),
    mean_dv = mean(overall_rating.c),
    sd_dv = sd(overall_rating.c),
    se_dv = se(overall_rating.c)
  )
```

```
## # A tibble: 2 x 5
##   partner.e     n   mean_dv     sd_dv      se_dv
##       <dbl> <int>     <dbl>     <dbl>      <dbl>
## 1      -0.5   210 0.3063844 0.4461867 0.03078981
## 2       0.5   111 0.4483861 0.5233885 0.04967784
```

Analyses H1p: Hormones (+ partnership status)

E + P + E*P: (+ partnership status)

Testing for effects of estradiol, progesterone, and their interaction on preferences

```
model_h1p_EP <- lmer(rating.c ~ estr.s * prog.s * context.e * partner.e + sess
ion_n +
                          (1 | oc_id) +
                          (0 + estr.s:prog.s:context.e || oc_id) +
                          (1 | block:oc_id) +
                          (0 + estr.s:prog.s:context.e|| block:oc_id) +
                          (1 | block:oc_id:date) +
                          (0 + context.e || block:oc_id:date),
                      data = filter(data_hormones_partner,
                                    face_sex==theFaceSex,
                                    manip==theManip),
                      REML = FALSE,
                     # use nelder-mead optimziation (as in lmer v < 1)
                     control = lmerControl(optimizer ="Nelder_Mead"))
summary_h1p_EP <- summary(model_h1p_EP)
mySummary(summary_h1p_EP)
```

```
## [[1]]
## block:oc_id:date      block:oc_id            oc_id
##             1829              389              321
##
## [[2]]
##
##
## rowname                                  Estimate   Std. Error          df   t v
alue   Pr(>|t|)  sig
## ---------------------------------  ---------  -----------  ---------  ----
----  ---------  ----
## (Intercept)                                0.426        0.031    448.353    13
.942      0.000  ***
## estr.s                                    -0.055        0.057   1524.518    -0
.951      0.342
## prog.s                                     0.005        0.046   1500.036     0
.111      0.912
## context.e                                  0.052        0.014   1824.334     3
.656      0.000  ***
## partner.e                                  0.145        0.056    331.663     2
.585      0.010  *
## session_n                                 -0.017        0.004    781.721    -3
.924      0.000  ***
## estr.s:prog.s                             -0.012        0.276   1560.202    -0
.043      0.966
## estr.s:context.e                           0.031        0.092   1828.769     0
.334      0.738
## prog.s:context.e                           0.026        0.078   1827.753     0
.336      0.737
## estr.s:partner.e                          -0.255        0.115   1520.185    -2
.224      0.026  *
## prog.s:partner.e                           0.068        0.092   1503.985     0
.741      0.459
## context.e:partner.e                        0.015        0.028   1824.334     0
.512      0.608
## estr.s:prog.s:context.e                    0.158        0.429   1843.423     0
.367      0.713
## estr.s:prog.s:partner.e                    0.611        0.552   1559.622     1
.106      0.269
## estr.s:context.e:partner.e                -0.029        0.185   1828.769    -0
.154      0.877
## prog.s:context.e:partner.e                -0.092        0.156   1827.753    -0
.589      0.556
## estr.s:prog.s:context.e:partner.e          0.043        0.858   1843.426     0
.050      0.960
```

```
confint(model_h1p_EP, method = confint_method) %>% as.data.frame() %>% rowname
s_to_column() %>% filter(!is.na(`2.5 %`))
```

```
##                               rowname       2.5 %       97.5 %
## 1                        (Intercept)  0.36606332  0.485817121
## 2                             estr.s -0.16710558  0.057956296
## 3                             prog.s -0.08524019  0.095487276
## 4                          context.e  0.02416887  0.080021388
## 5                          partner.e  0.03509114  0.255149907
## 6                          session_n -0.02564537 -0.008561301
## 7                      estr.s:prog.s -0.55299537  0.529445071
## 8                   estr.s:context.e -0.15037506  0.212213002
## 9                   prog.s:context.e -0.12662359  0.178998182
## 10                  estr.s:partner.e -0.47973940 -0.030248324
## 11                  prog.s:partner.e -0.11228872  0.248742773
## 12               context.e:partner.e -0.04124848  0.070456548
## 13           estr.s:prog.s:context.e -0.68329355  0.998416024
## 14           estr.s:prog.s:partner.e -0.47164324  1.693129701
## 15        estr.s:context.e:partner.e -0.39115893  0.334017108
## 16        prog.s:context.e:partner.e -0.39743528  0.213808085
## 17 estr.s:prog.s:context.e:partner.e -1.63840366  1.725011241
```

E + P + E*P: (single women only to interpret interaction)

```
model_h1p_EP_single <- lmer(rating.c ~ estr.s * prog.s * context.e + session_n
+
                          (1 | oc_id) +
                          (0 + estr.s:prog.s:context.e || oc_id) +
                          (1 | block:oc_id) +
                          (0 + estr.s:prog.s:context.e|| block:oc_id) +
                          (1 | block:oc_id:date) +
                          (0 + context.e || block:oc_id:date),
                      data = filter(data_hormones_partner,
                                    face_sex==theFaceSex,
                                    manip==theManip,
                                    partner.e==-0.5),
                      REML = FALSE)
```

```
## Warning in checkConv(attr(opt, "derivs"), opt$par, ctrl = control$checkConv
, : Model is nearly unidentifiable: large eigenvalue ratio
##  - Rescale variables?
```

```
summary_h1p_EP_single <- summary(model_h1p_EP_single)
mySummary(summary_h1p_EP_single)
```

```
## [[1]]
## block:oc_id:date      block:oc_id           oc_id
##             1213              257             210
##
## [[2]]
##
##
## rowname                      Estimate   Std. Error         df   t value   Pr(
>|t|)  sig
## ------------------------  ---------  -----------  ---------  --------  ----
-----  ----
## (Intercept)                  0.365        0.034    306.784    10.772
0.000  ***
## estr.s                       0.068        0.065   1014.804     1.051
0.294
## prog.s                      -0.025        0.059   1001.386    -0.416
0.677
## context.e                    0.045        0.017   1194.962     2.694
0.007  **
## session_n                   -0.021        0.005    470.549    -4.094
0.000  ***
## estr.s:prog.s               -0.322        0.333   1033.324    -0.967
0.334
## estr.s:context.e             0.044        0.109   1183.279     0.405
0.686
## prog.s:context.e             0.088        0.101   1012.040     0.876
0.381
## estr.s:prog.s:context.e      0.177        0.610     46.506     0.290
0.773
```

```
confint(model_h1p_EP_single, method = confint_method) %>% as.data.frame() %>%
rownames_to_column() %>% filter(!is.na(`2.5 %`))
```

```
##                  rowname       2.5 %      97.5 %
## 1            (Intercept)  0.29837192  0.43109858
## 2                 estr.s -0.05920280  0.19611554
## 3                 prog.s -0.13997796  0.09094831
## 4              context.e  0.01213906  0.07700589
## 5              session_n -0.03144196 -0.01108290
## 6          estr.s:prog.s -0.97470177  0.33077355
## 7       estr.s:context.e -0.16981893  0.25819050
## 8       prog.s:context.e -0.10915732  0.28576098
## 9 estr.s:prog.s:context.e -1.01941670  1.37332738
```

E + P + E*P: (partnered women only to interpret interaction)

```
model_h1p_EP_partnered<- lmer(rating.c ~ estr.s * prog.s * context.e + session
_n +
                          (1 | oc_id) +
                          (0 + estr.s:prog.s:context.e || oc_id) +
                          (1 | block:oc_id) +
                          (0 + estr.s:prog.s:context.e|| block:oc_id) +
                          (1 | block:oc_id:date) +
                          (0 + context.e || block:oc_id:date),
                      data = filter(data_hormones_partner,
                                    face_sex==theFaceSex,
                                    manip==theManip,
                                    partner.e==0.5),
                      REML = FALSE)
summary_h1p_EP_partnered <- summary(model_h1p_EP_partnered)
mySummary(summary_h1p_EP_partnered)
```

```
## [[1]]
## block:oc_id:date      block:oc_id            oc_id
##              616              132              111
##
## [[2]]
##
##
## rowname                    Estimate   Std. Error        df   t value   Pr(>
|t|)  sig
## ------------------------  ---------  -----------  --------  --------  -----
----  ----
## (Intercept)                   0.476        0.055   156.368     8.605      0
.000  ***
## estr.s                       -0.184        0.097   509.662    -1.895      0
.059  +
## prog.s                        0.037        0.072   499.467     0.514      0
.608
## context.e                     0.059        0.023   611.615     2.546      0
.011  *
## session_n                    -0.009        0.008   310.422    -1.128      0
.260
## estr.s:prog.s                 0.314        0.449   519.964     0.700      0
.484
## estr.s:context.e              0.017        0.151   611.925     0.115      0
.909
## prog.s:context.e             -0.020        0.121   613.555    -0.162      0
.871
## estr.s:prog.s:context.e       0.187        0.685   622.045     0.272      0
.785
```

```
confint(model_h1p_EP_partnered, method = confint_method) %>% as.data.frame() %
>% rownames_to_column() %>% filter(!is.na(`2.5 %`))
```

```
##                      rowname       2.5 %      97.5 %
## 1              (Intercept)  0.36739476 0.584110357
## 2                   estr.s -0.37352261 0.006266024
## 3                   prog.s -0.10441485 0.178561485
## 4                context.e  0.01368495 0.105252094
## 5                session_n -0.02454124 0.006608364
## 6            estr.s:prog.s -0.56618316 1.194798314
## 7         estr.s:context.e -0.27828146 0.312884884
## 8         prog.s:context.e -0.25652139 0.217263246
## 9 estr.s:prog.s:context.e -1.15517448 1.528229097
```

Note that the non-significant effect of estradiol (estimate = -0.18, p = .064) is in the opposite direction to what would be predicted from previous work reporting positive effects of estradiol on preferences.

E + P + EPratio: (+ partnership status)

```
model_h1p_EP_EPratio <- lmer(rating.c ~ estr.s * context.e * partner.e +
                                                 prog.s * context.e * partner.
e +
                                                 ep_ratio.s * context.e * part
ner.e + session_n +
                          (1 | oc_id) +
                          (0 + estr.s:context.e + prog.s:context.e + ep_ratio.
s:context.e || oc_id) +
                          (1 | block:oc_id) +
                          (0 + estr.s:context.e + prog.s:context.e + ep_ratio.
s:context.e|| block:oc_id) +
                          (1 | block:oc_id:date) +
                          (0 + context.e || block:oc_id:date),
                      data = filter(data_hormones_partner,
                                    face_sex==theFaceSex,
                                    manip==theManip),
                      REML = FALSE)
summary_h1p_EP_EPratio <- summary(model_h1p_EP_EPratio)
mySummary(summary_h1p_EP_EPratio)
```

```
## [[1]]
## block:oc_id:date      block:oc_id           oc_id
##             1829              389             321
##
## [[2]]
##
##
## rowname                            Estimate   Std. Error         df   t valu
e   Pr(>|t|)  sig
## ------------------------------  ---------  -----------  ---------  -------
-  ---------  ----
## (Intercept)                         0.426        0.030    442.881    13.99
0      0.000  ***
## estr.s                             -0.047        0.058   1525.690    -0.79
7      0.426
## context.e                           0.053        0.014   1825.327     3.90
1      0.000  ***
## partner.e                           0.151        0.056    327.020     2.69
2      0.007  **
## prog.s                             -0.012        0.052   1490.862    -0.23
3      0.816
## ep_ratio.s                         -0.006        0.027   1487.415    -0.24
0      0.811
## session_n                          -0.017        0.004    785.285    -3.94
3      0.000  ***
## estr.s:context.e                    0.040        0.094   1827.758     0.42
3      0.672
## estr.s:partner.e                   -0.211        0.117   1522.741    -1.80
5      0.071  +
## context.e:partner.e                 0.015        0.027   1825.327     0.54
0      0.589
## context.e:prog.s                    0.002        0.089   1827.927     0.02
7      0.979
## partner.e:prog.s                   -0.013        0.105   1493.486    -0.12
2      0.903
## context.e:ep_ratio.s               -0.031        0.046   1822.168    -0.67
1      0.502
## partner.e:ep_ratio.s               -0.094        0.054   1487.687    -1.73
0      0.084  +
## estr.s:context.e:partner.e         -0.053        0.189   1827.758    -0.28
3      0.777
## context.e:partner.e:prog.s         -0.036        0.179   1827.927    -0.20
2      0.840
## context.e:partner.e:ep_ratio.s      0.068        0.093   1822.167     0.73
4      0.463
```

```
confint(model_h1p_EP_EPratio, method = confint_method) %>% as.data.frame() %>%
rownames_to_column() %>% filter(!is.na(`2.5 %`))
```

```
##                            rowname       2.5 %       97.5 %
## 1                      (Intercept)  0.36631251  0.485675874
## 2                           estr.s -0.16091873  0.067898350
## 3                        context.e  0.02660868  0.080334099
## 4                        partner.e  0.04098187  0.260297814
## 5                           prog.s -0.11479394  0.090443965
## 6                       ep_ratio.s -0.05955255  0.046574593
## 7                        session_n -0.02571215 -0.008637281
## 8                 estr.s:context.e -0.14512286  0.225040882
## 9                 estr.s:partner.e -0.43908057  0.018015728
## 10             context.e:partner.e -0.03891104  0.068539748
## 11                context.e:prog.s -0.17276633  0.177511198
## 12                partner.e:prog.s -0.21787043  0.192430750
## 13            context.e:ep_ratio.s -0.12187774  0.059697382
## 14            partner.e:ep_ratio.s -0.19979771  0.012442808
## 15      estr.s:context.e:partner.e -0.42362610  0.316701350
## 16      context.e:partner.e:prog.s -0.38635090  0.314204150
## 17 context.e:partner.e:ep_ratio.s -0.11360621  0.249544098
```

T + C: (+ partnership status)

Testing for effects of testosterone and coritsol on preferences

```
model_h1p_TC <- lmer(rating.c ~ test.s*context.e*partner.e +
                                         cort.s*context.e*partner.e + session_
n +
                          (1 | oc_id) +
                          (0 + test.s:context.e + cort.s:context.e || oc_id) +
                          (1 | block:oc_id) +
                          (0 + test.s:context.e + cort.s:context.e|| block:oc_
id) +
                          (1 | block:oc_id:date) +
                          (0 + context.e || block:oc_id:date),
                      data = filter(data_hormones_partner,
                                    face_sex==theFaceSex,
                                    manip==theManip),
                      REML = FALSE)
summary_h1p_TC <- summary(model_h1p_TC)
mySummary(summary_h1p_TC)
```

```
## [[1]]
## block:oc_id:date      block:oc_id           oc_id
##             1846              388             320
##
## [[2]]
##
##
## rowname                       Estimate   Std. Error         df   t value
Pr(>|t|)  sig
## ---------------------------  ---------  -----------  ---------  --------  -
--------  ----
## (Intercept)                      0.424        0.030    432.502    14.022
0.000  ***
## test.s                          -0.009        0.054   1532.447    -0.160
0.873
## context.e                        0.054        0.014   1842.428     3.989
0.000  ***
## partner.e                        0.146        0.056    323.959     2.619
0.009  **
## cort.s                           0.039        0.042   1528.308     0.925
0.355
## session_n                       -0.016        0.004    612.761    -3.823
0.000  ***
## test.s:context.e                 0.037        0.090   1840.655     0.408
0.684
## test.s:partner.e                -0.061        0.108   1532.345    -0.568
0.570
## context.e:partner.e              0.009        0.027   1842.428     0.312
0.755
## context.e:cort.s                -0.016        0.070   1839.047    -0.235
0.814
## partner.e:cort.s                 0.037        0.085   1527.107     0.440
0.660
## test.s:context.e:partner.e       0.007        0.181   1840.654     0.038
0.970
## context.e:partner.e:cort.s       0.004        0.139   1839.048     0.032
0.974
```

```
confint(model_h1p_TC, method = confint_method) %>% as.data.frame() %>% rowname
s_to_column() %>% filter(!is.na(`2.5 %`))
```

```
##                         rowname       2.5 %       97.5 %
## 1                   (Intercept)  0.36475511  0.483289751
## 2                        test.s -0.11476123  0.097468978
## 3                     context.e  0.02765686  0.081094792
## 4                     partner.e  0.03673752  0.255209685
## 5                        cort.s -0.04385607  0.122297045
## 6                     session_n -0.02450491 -0.007893281
## 7              test.s:context.e -0.14046155  0.214214589
## 8              test.s:partner.e -0.27367579  0.150766690
## 9           context.e:partner.e -0.04493114  0.061944661
## 10             context.e:cort.s -0.15283413  0.120149187
## 11             partner.e:cort.s -0.12882631  0.203503403
## 12 test.s:context.e:partner.e -0.34783333  0.361519241
## 13 context.e:partner.e:cort.s -0.26852653  0.277440092
```

T + C + T*C: (+ partnership status)

Testing for effects of testosterone and coritsol plus their interaction on preferences

```
# Converges when we model random slopes for individual predictors and the inte
raction on oc_id
model_h1p_TCi <- lmer(rating.c ~ test.s * context.e * cort.s *partner.e +
                          (1 | oc_id) +
                          (0 + test.s*cort.s*context.e || oc_id) +
                          (1 | block:oc_id) +
                          (0 + test.s*cort.s*context.e|| block:oc_id) +
                          (1 | block:oc_id:date) +
                          (0 + context.e || block:oc_id:date),
                     data = filter(data_hormones_partner,
                                   face_sex==theFaceSex,
                                   manip==theManip),
                     REML = FALSE)
summary_h1p_TCi <- summary(model_h1p_TCi)
mySummary(summary_h1p_TCi)
```

```
## [[1]]
## block:oc_id:date      block:oc_id            oc_id
##             1846              388               320
##
## [[2]]
##
##
## rowname                              Estimate   Std. Error          df    t v
alue   Pr(>|t|)  sig
## ---------------------------------  ---------  -----------  ---------  ----
----  ---------  ----
## (Intercept)                              0.373        0.028    331.790    13
.362      0.000  ***
## test.s                                  -0.003        0.056    911.345    -0
.052      0.959
## context.e                                0.053        0.019    349.311     2
.819      0.005  **
## cort.s                                   0.025        0.049     19.986     0
.504      0.620
## partner.e                                0.143        0.056    331.790     2
.555      0.011  *
## test.s:context.e                         0.029        0.084   1500.097     0
.345      0.730
## test.s:cort.s                            0.467        0.248    128.086     1
.882      0.062  +
## context.e:cort.s                        -0.010        0.067   1058.835    -0
.147      0.883
## test.s:partner.e                        -0.034        0.111    911.345    -0
.307      0.759
## context.e:partner.e                      0.005        0.038    349.311     0
.137      0.891
## cort.s:partner.e                         0.008        0.098     19.986     0
.080      0.937
## test.s:context.e:cort.s                 -0.079        0.344    119.267    -0
.229      0.819
## test.s:context.e:partner.e               0.028        0.169   1500.097     0
.168      0.867
## test.s:cort.s:partner.e                  0.503        0.496    128.086     1
.014      0.312
## context.e:cort.s:partner.e              -0.013        0.135   1058.835    -0
.097      0.923
## test.s:context.e:cort.s:partner.e        0.201        0.687    119.267     0
.292      0.771
```

```
confint(model_h1p_TCi, method = confint_method) %>% as.data.frame() %>% rownam
es_to_column() %>% filter(!is.na(`2.5 %`))
```

```
##                                rowname       2.5 %     97.5 %
## 1                          (Intercept)  0.31861762 0.42815523
## 2                               test.s -0.11170927 0.10597994
## 3                            context.e  0.01613966 0.08982415
## 4                               cort.s -0.07146086 0.12096978
## 5                            partner.e  0.03328075 0.25235597
## 6                     test.s:context.e -0.13619310 0.19437599
## 7                        test.s:cort.s -0.01938666 0.95301568
## 8                     context.e:cort.s -0.14188202 0.12209433
## 9                     test.s:partner.e -0.25175183 0.18362660
## 10                 context.e:partner.e -0.06851595 0.07885303
## 11                    cort.s:partner.e -0.18459377 0.20026750
## 12             test.s:context.e:cort.s -0.75224120 0.59501725
## 13          test.s:context.e:partner.e -0.30227308 0.35886510
## 14             test.s:cort.s:partner.e -0.46928547 1.47551921
## 15          context.e:cort.s:partner.e -0.27705667 0.25089603
## 16 test.s:context.e:cort.s:partner.e -1.14666416 1.54785273
```

Analyses H1ps: Hormones (+ session order, + partnership status)

E + P + E*P: (+ session order, + partnership status)

Testing for effects of estradiol, progesterone, and their interaction on preferences

```
model_h1_EP_partner_s <- lmer(rating.c ~ estr.s * prog.s * context.e * partner
.e + session_n +
                          (1 | oc_id) +
                          (0 + estr.s:prog.s:context.e || oc_id) +
                          (1 | block:oc_id) +
                          (0 + estr.s:prog.s:context.e|| block:oc_id) +
                          (1 | block:oc_id:date) +
                          (0 + context.e || block:oc_id:date),
                      data = filter(data_hormones_partner,
                                    face_sex==theFaceSex,
                                    manip==theManip),
                      REML = FALSE,
                     # use nelder-mead optimziation (as in lmer v < 1)
                     control = lmerControl(optimizer ="Nelder_Mead"))
summary_h1_EP_partner_s <- summary(model_h1_EP_partner_s)
mySummary(summary_h1_EP_partner_s)
```

```
## [[1]]
## block:oc_id:date      block:oc_id           oc_id
##             1829              389              321
##
## [[2]]
##
##
## rownames                                  Estimate   Std. Error          df   t v
alue   Pr(>|t|)  sig
## ----------------------------------  ---------  -----------  ---------  ----
----  ---------  ----
## (Intercept)                               0.426        0.031     448.353    13
.942      0.000  ***
## estr.s                                   -0.055        0.057    1524.518    -0
.951      0.342
## prog.s                                    0.005        0.046    1500.036     0
.111      0.912
## context.e                                 0.052        0.014    1824.334     3
.656      0.000  ***
## partner.e                                 0.145        0.056     331.663     2
.585      0.010  *
## session_n                                -0.017        0.004     781.721    -3
.924      0.000  ***
## estr.s:prog.s                            -0.012        0.276    1560.202    -0
.043      0.966
## estr.s:context.e                          0.031        0.092    1828.769     0
.334      0.738
## prog.s:context.e                          0.026        0.078    1827.753     0
.336      0.737
## estr.s:partner.e                         -0.255        0.115    1520.185    -2
.224      0.026  *
## prog.s:partner.e                          0.068        0.092    1503.985     0
.741      0.459
## context.e:partner.e                       0.015        0.028    1824.334     0
.512      0.608
## estr.s:prog.s:context.e                   0.158        0.429    1843.423     0
.367      0.713
## estr.s:prog.s:partner.e                   0.611        0.552    1559.622     1
.106      0.269
## estr.s:context.e:partner.e               -0.029        0.185    1828.769    -0
.154      0.877
## prog.s:context.e:partner.e               -0.092        0.156    1827.753    -0
.589      0.556
## estr.s:prog.s:context.e:partner.e         0.043        0.858    1843.426     0
.050      0.960
```

```
confint(model_h1_EP_partner_s, method = confint_method) %>% as.data.frame() %>
% rownames_to_column() %>% filter(!is.na(`2.5 %`))
```

```
##                               rowname       2.5 %       97.5 %
## 1                         (Intercept)  0.36606332  0.485817121
## 2                              estr.s -0.16710558  0.057956296
## 3                              prog.s -0.08524019  0.095487276
## 4                           context.e  0.02416887  0.080021388
## 5                           partner.e  0.03509114  0.255149907
## 6                           session_n -0.02564537 -0.008561301
## 7                       estr.s:prog.s -0.55299537  0.529445071
## 8                    estr.s:context.e -0.15037506  0.212213002
## 9                    prog.s:context.e -0.12662359  0.178998182
## 10                   estr.s:partner.e -0.47973940 -0.030248324
## 11                   prog.s:partner.e -0.11228872  0.248742773
## 12                context.e:partner.e -0.04124848  0.070456548
## 13            estr.s:prog.s:context.e -0.68329355  0.998416024
## 14            estr.s:prog.s:partner.e -0.47164324  1.693129701
## 15         estr.s:context.e:partner.e -0.39115893  0.334017108
## 16         prog.s:context.e:partner.e -0.39743528  0.213808085
## 17 estr.s:prog.s:context.e:partner.e -1.63840366  1.725011241
```

E + P + E*P: (single women only to interpret interaction)

```
model_h1_EP_partner_single_s <- lmer(rating.c ~ estr.s * prog.s * context.e +
session_n +
                          (1 | oc_id) +
                          (0 + estr.s:prog.s:context.e || oc_id) +
                          (1 | block:oc_id) +
                          (0 + estr.s:prog.s:context.e|| block:oc_id) +
                          (1 | block:oc_id:date) +
                          (0 + context.e || block:oc_id:date),
                      data = filter(data_hormones_partner,
                                    face_sex==theFaceSex,
                                    manip==theManip,
                                    partner.e==-0.5),
                      REML = FALSE)
```

```
## Warning in checkConv(attr(opt, "derivs"), opt$par, ctrl = control$checkConv
, : Model is nearly unidentifiable: large eigenvalue ratio
##  - Rescale variables?
```

```
summary_h1_EP_partner_single_s <- summary(model_h1_EP_partner_single_s)
mySummary(summary_h1_EP_partner_single_s)
```

```
## [[1]]
## block:oc_id:date      block:oc_id            oc_id
##             1213              257              210
##
## [[2]]
##
##
## rowname                     Estimate   Std. Error         df   t value   Pr(
>|t|)  sig
## ------------------------  ---------  -----------  ---------  --------  ----
-----  ----
## (Intercept)                   0.365        0.034    306.784    10.772
0.000  ***
## estr.s                        0.068        0.065   1014.804     1.051
0.294
## prog.s                       -0.025        0.059   1001.386    -0.416
0.677
## context.e                     0.045        0.017   1194.962     2.694
0.007  **
## session_n                    -0.021        0.005    470.549    -4.094
0.000  ***
## estr.s:prog.s                -0.322        0.333   1033.324    -0.967
0.334
## estr.s:context.e              0.044        0.109   1183.279     0.405
0.686
## prog.s:context.e              0.088        0.101   1012.040     0.876
0.381
## estr.s:prog.s:context.e       0.177        0.610     46.506     0.290
0.773
```

```
confint(model_h1_EP_partner_single_s, method = confint_method) %>% as.data.fra
me() %>% rownames_to_column() %>% filter(!is.na(`2.5 %`))
```

```
##                   rowname       2.5 %      97.5 %
## 1             (Intercept)  0.29837192  0.43109858
## 2                  estr.s -0.05920280  0.19611554
## 3                  prog.s -0.13997796  0.09094831
## 4               context.e  0.01213906  0.07700589
## 5               session_n -0.03144196 -0.01108290
## 6           estr.s:prog.s -0.97470177  0.33077355
## 7        estr.s:context.e -0.16981893  0.25819050
## 8        prog.s:context.e -0.10915732  0.28576098
## 9 estr.s:prog.s:context.e -1.01941670  1.37332738
```

E + P + E*P: (partnered women only to interpret interaction)

```
model_h1_EP_partner_partnered_s <- lmer(rating.c ~ estr.s * prog.s * context.e
+ session_n +
                          (1 | oc_id) +
                          (0 + estr.s:prog.s:context.e || oc_id) +
                          (1 | block:oc_id) +
                          (0 + estr.s:prog.s:context.e|| block:oc_id) +
                          (1 | block:oc_id:date) +
                          (0 + context.e || block:oc_id:date),
                      data = filter(data_hormones_partner,
                                    face_sex==theFaceSex,
                                    manip==theManip,
                                    partner.e==0.5),
                      REML = FALSE)
summary_h1_EP_partner_partnered_s <- summary(model_h1_EP_partner_partnered_s)
mySummary(summary_h1_EP_partner_partnered_s)
```

```
## [[1]]
## block:oc_id:date      block:oc_id            oc_id
##              616              132              111
##
## [[2]]
##
##
## rowname                    Estimate   Std. Error        df   t value   Pr(>
|t|)  sig
## -----------------------  ---------  -----------  --------  --------  -----
----  ----
## (Intercept)                  0.476        0.055   156.368     8.605       0
.000  ***
## estr.s                      -0.184        0.097   509.662    -1.895       0
.059  +
## prog.s                       0.037        0.072   499.467     0.514       0
.608
## context.e                    0.059        0.023   611.615     2.546       0
.011  *
## session_n                   -0.009        0.008   310.422    -1.128       0
.260
## estr.s:prog.s                0.314        0.449   519.964     0.700       0
.484
## estr.s:context.e             0.017        0.151   611.925     0.115       0
.909
## prog.s:context.e            -0.020        0.121   613.555    -0.162       0
.871
## estr.s:prog.s:context.e      0.187        0.685   622.045     0.272       0
.785
```

```
confint(model_h1_EP_partner_partnered_s, method = confint_method) %>% as.data.
frame() %>% rownames_to_column() %>% filter(!is.na(`2.5 %`))
```

```
##                     rowname       2.5 %      97.5 %
## 1              (Intercept)  0.36739476 0.584110357
## 2                   estr.s -0.37352261 0.006266024
## 3                   prog.s -0.10441485 0.178561485
## 4                context.e  0.01368495 0.105252094
## 5                session_n -0.02454124 0.006608364
## 6            estr.s:prog.s -0.56618316 1.194798314
## 7         estr.s:context.e -0.27828146 0.312884884
## 8         prog.s:context.e -0.25652139 0.217263246
## 9 estr.s:prog.s:context.e -1.15517448 1.528229097
```

Note that the non-significant effect of estradiol (estimate = -0.18, p = .064) is in the opposite direction to what would be predicted from previous work reporting positive effects of estradiol.

E + P + EPratio: (+ session order, + partnership status)

```
model_h1p_EP_EPratio_s <- lmer(rating.c ~ estr.s * context.e * partner.e +
                                                 prog.s * context.e * partner.
e +
                                                 ep_ratio.s * context.e * part
ner.e + session_n +
                          (1 | oc_id) +
                          (0 + estr.s:context.e + prog.s:context.e + ep_ratio.
s:context.e || oc_id) +
                          (1 | block:oc_id) +
                          (0 + estr.s:context.e + prog.s:context.e + ep_ratio.
s:context.e|| block:oc_id) +
                          (1 | block:oc_id:date) +
                          (0 + context.e || block:oc_id:date),
                      data = filter(data_hormones_partner,
                                    face_sex==theFaceSex,
                                    manip==theManip),
                      REML = FALSE)
summary_h1p_EP_EPratio_s <- summary(model_h1p_EP_EPratio_s)
mySummary(summary_h1p_EP_EPratio_s)
```

```
## [[1]]
## block:oc_id:date      block:oc_id           oc_id
##             1829              389             321
##
## [[2]]
##
##
## rowname                              Estimate   Std. Error          df   t valu
e   Pr(>|t|)  sig
## ------------------------------  ---------  -----------  ---------  -------
-  ---------  ----
## (Intercept)                          0.426        0.030    442.881    13.99
0      0.000  ***
## estr.s                              -0.047        0.058   1525.690    -0.79
7      0.426
## context.e                            0.053        0.014   1825.327     3.90
1      0.000  ***
## partner.e                            0.151        0.056    327.020     2.69
2      0.007  **
## prog.s                              -0.012        0.052   1490.862    -0.23
3      0.816
## ep_ratio.s                          -0.006        0.027   1487.415    -0.24
0      0.811
## session_n                           -0.017        0.004    785.285    -3.94
3      0.000  ***
## estr.s:context.e                     0.040        0.094   1827.758     0.42
3      0.672
## estr.s:partner.e                    -0.211        0.117   1522.741    -1.80
5      0.071  +
## context.e:partner.e                  0.015        0.027   1825.327     0.54
0      0.589
## context.e:prog.s                     0.002        0.089   1827.927     0.02
7      0.979
## partner.e:prog.s                    -0.013        0.105   1493.486    -0.12
2      0.903
## context.e:ep_ratio.s                -0.031        0.046   1822.168    -0.67
1      0.502
## partner.e:ep_ratio.s                -0.094        0.054   1487.687    -1.73
0      0.084  +
## estr.s:context.e:partner.e          -0.053        0.189   1827.758    -0.28
3      0.777
## context.e:partner.e:prog.s          -0.036        0.179   1827.927    -0.20
2      0.840
## context.e:partner.e:ep_ratio.s       0.068        0.093   1822.167     0.73
4      0.463
```

```
confint(model_h1p_EP_EPratio_s, method = confint_method) %>% as.data.frame() %
>% rownames_to_column() %>% filter(!is.na(`2.5 %`))
```

```
##                                rowname       2.5 %       97.5 %
## 1                          (Intercept)  0.36631251  0.485675874
## 2                               estr.s -0.16091873  0.067898350
## 3                            context.e  0.02660868  0.080334099
## 4                            partner.e  0.04098187  0.260297814
## 5                               prog.s -0.11479394  0.090443965
## 6                           ep_ratio.s -0.05955255  0.046574593
## 7                            session_n -0.02571215 -0.008637281
## 8                   estr.s:context.e -0.14512286  0.225040882
## 9                   estr.s:partner.e -0.43908057  0.018015728
## 10             context.e:partner.e -0.03891104  0.068539748
## 11                  context.e:prog.s -0.17276633  0.177511198
## 12                  partner.e:prog.s -0.21787043  0.192430750
## 13              context.e:ep_ratio.s -0.12187774  0.059697382
## 14              partner.e:ep_ratio.s -0.19979771  0.012442808
## 15        estr.s:context.e:partner.e -0.42362610  0.316701350
## 16        context.e:partner.e:prog.s -0.38635090  0.314204150
## 17 context.e:partner.e:ep_ratio.s -0.11360621  0.249544098
```

T + C: (+ session order, + partnership status)

Testing for effects of testosterone and coritsol on preferences

```
model_h1p_TC_s <- lmer(rating.c ~ test.s*context.e*partner.e +
                                          cort.s*context.e*partner.e + session_
n +
                          (1 | oc_id) +
                          (0 + test.s:context.e + cort.s:context.e || oc_id) +
                          (1 | block:oc_id) +
                          (0 + test.s:context.e + cort.s:context.e|| block:oc_
id) +
                          (1 | block:oc_id:date) +
                          (0 + context.e || block:oc_id:date),
                      data = filter(data_hormones_partner,
                                    face_sex==theFaceSex,
                                    manip==theManip),
                      REML = FALSE)
summary_h1p_TC_s <- summary(model_h1p_TC_s)
mySummary(summary_h1p_TC_s)
```

```
## [[1]]
## block:oc_id:date      block:oc_id            oc_id
##             1846              388              320
##
## [[2]]
##
##
## rowname                        Estimate   Std. Error         df   t value
Pr(>|t|)  sig
## ---------------------------  ---------  -----------  ---------  --------  -
--------  ----
## (Intercept)                       0.424        0.030    432.502    14.022
0.000  ***
## test.s                           -0.009        0.054   1532.447    -0.160
0.873
## context.e                         0.054        0.014   1842.428     3.989
0.000  ***
## partner.e                         0.146        0.056    323.959     2.619
0.009  **
## cort.s                            0.039        0.042   1528.308     0.925
0.355
## session_n                        -0.016        0.004    612.761    -3.823
0.000  ***
## test.s:context.e                  0.037        0.090   1840.655     0.408
0.684
## test.s:partner.e                 -0.061        0.108   1532.345    -0.568
0.570
## context.e:partner.e               0.009        0.027   1842.428     0.312
0.755
## context.e:cort.s                 -0.016        0.070   1839.047    -0.235
0.814
## partner.e:cort.s                  0.037        0.085   1527.107     0.440
0.660
## test.s:context.e:partner.e        0.007        0.181   1840.654     0.038
0.970
## context.e:partner.e:cort.s        0.004        0.139   1839.048     0.032
0.974
```

```
confint(model_h1p_TC_s, method = confint_method) %>% as.data.frame() %>% rowna
mes_to_column() %>% filter(!is.na(`2.5 %`))
```

```
##                         rowname       2.5 %       97.5 %
## 1                  (Intercept)  0.36475511  0.483289751
## 2                       test.s -0.11476123  0.097468978
## 3                    context.e  0.02765686  0.081094792
## 4                    partner.e  0.03673752  0.255209685
## 5                       cort.s -0.04385607  0.122297045
## 6                    session_n -0.02450491 -0.007893281
## 7             test.s:context.e -0.14046155  0.214214589
## 8             test.s:partner.e -0.27367579  0.150766690
## 9          context.e:partner.e -0.04493114  0.061944661
## 10            context.e:cort.s -0.15283413  0.120149187
## 11            partner.e:cort.s -0.12882631  0.203503403
## 12 test.s:context.e:partner.e -0.34783333  0.361519241
## 13 context.e:partner.e:cort.s -0.26852653  0.277440092
```

T + C + T*C: (+ session order, + partnership status)

```
model_h1p_TCi_s <- lmer(rating.c ~ cort.s*test.s*context.e*partner.e + session
_n +
                          (1 | oc_id) +
                          (0 + test.s:cort.s:context.e || oc_id) +
                          (1 | block:oc_id) +
                          (0 + test.s:cort.s:context.e|| block:oc_id) +
                          (1 | block:oc_id:date) +
                          (0 + context.e || block:oc_id:date),
                      data = filter(data_hormones_partner,
                                    face_sex==theFaceSex,
                                    manip==theManip),
                      REML = FALSE)
summary_h1p_TCi_s <- summary(model_h1p_TCi_s)
mySummary(summary_h1p_TCi_s)
```

```
## [[1]]
## block:oc_id:date      block:oc_id           oc_id
##             1846              388             320
##
## [[2]]
##
##
## rowname                              Estimate   Std. Error         df   t v
alue   Pr(>|t|)  sig
## ---------------------------------  ---------  -----------  ---------  ----
----  ---------  ----
## (Intercept)                            0.419        0.030    439.928    13
.801      0.000  ***
## cort.s                                 0.025        0.043   1529.942     0
.586      0.558
## test.s                                -0.004        0.054   1532.392    -0
.072      0.943
## context.e                              0.056        0.014   1840.561     3
.850      0.000  ***
## partner.e                              0.141        0.056    331.147     2
.518      0.012  *
## session_n                             -0.016        0.004    612.014    -3
.801      0.000  ***
## cort.s:test.s                          0.375        0.221   1595.136     1
.702      0.089  +
## cort.s:context.e                      -0.010        0.072   1111.058    -0
.138      0.890
## test.s:context.e                       0.037        0.091   1756.212     0
.401      0.688
## cort.s:partner.e                       0.025        0.087   1529.013     0
.289      0.773
## test.s:partner.e                      -0.049        0.108   1532.276    -0
.453      0.650
## context.e:partner.e                    0.006        0.029   1840.561     0
.209      0.835
## cort.s:test.s:context.e               -0.139        0.374     67.938    -0
.370      0.712
## cort.s:test.s:partner.e                0.483        0.441   1594.468     1
.096      0.273
## cort.s:context.e:partner.e            -0.003        0.145   1111.058    -0
.019      0.985
## test.s:context.e:partner.e            -0.015        0.182   1756.212    -0
.081      0.936
## cort.s:test.s:context.e:partner.e      0.086        0.748     67.938     0
.114      0.909
```

```
confint(model_h1p_TCi_s, method = confint_method) %>% as.data.frame() %>% rown
ames_to_column() %>% filter(!is.na(`2.5 %`))
```

```
##                                rowname        2.5 %       97.5 %
## 1                         (Intercept)  0.35955593  0.478590412
## 2                              cort.s -0.05958307  0.110410402
## 3                              test.s -0.11012168  0.102341259
## 4                           context.e  0.02728460  0.083879674
## 5                           partner.e  0.03124191  0.250749902
## 6                           session_n -0.02439409 -0.007795043
## 7                       cort.s:test.s -0.05678580  0.807712891
## 8                    cort.s:context.e -0.15196333  0.131962503
## 9                    test.s:context.e -0.14199738  0.215049877
## 10                   cort.s:partner.e -0.14496449  0.195036104
## 11                   test.s:partner.e -0.26160416  0.163306340
## 12                context.e:partner.e -0.05056463  0.062625491
## 13            cort.s:test.s:context.e -0.87196470  0.594855233
## 14            cort.s:test.s:partner.e -0.38126739  1.347856324
## 15         cort.s:context.e:partner.e -0.28670343  0.281148225
## 16         test.s:context.e:partner.e -0.37174946  0.342345359
## 17 cort.s:test.s:context.e:partner.e -1.38122168  1.552418569
```

Hypothesis 2

Do women not using hormonal contraceptives show stronger preferences than women using the combined oral contraceptive pill?

One of these women was excluded from analyses because she did not complete any male face preference tests.

Descriptive stats: data_between

```
# create mean DV for all ratings by oc_id
stats_overall <- filter(data_between, face_sex==theFaceSex, manip==theManip) %
>%
  group_by(oc_id) %>%
  summarise(
    overall_rating.c = mean(rating.c)
  ) %>%
  ungroup() %>%
  group_by() %>%
  summarise(
    context = "overall",
    pill.e = "overall",
    n= n_distinct(oc_id),
    mean_dv = mean(overall_rating.c),
    sd_dv = sd(overall_rating.c),
    se_dv = se(overall_rating.c)
  )

# create mean DV splitting by context
stats_context <- filter(data_between, face_sex==theFaceSex, manip==theManip) %
>%
  group_by(oc_id, context) %>%
  summarise(
    context_rating.c = mean(rating.c)
  ) %>%
  group_by(context) %>%
  summarise(
    pill.e = "overall",
    n= n_distinct(oc_id),
    mean_dv = mean(context_rating.c),
    sd_dv = sd(context_rating.c),
    se_dv = se(context_rating.c)
  )

# stats by pill.e
stats_pill <- filter(data_between, face_sex==theFaceSex, manip==theManip) %>%
  group_by(oc_id, pill.e) %>%
  summarise(
    overall_rating.c = mean(rating.c)
  ) %>%
  ungroup() %>%
  group_by(pill.e) %>%
  summarise(
    context = "overall",
    n= n_distinct(oc_id),
    mean_dv = mean(overall_rating.c),
    sd_dv = sd(overall_rating.c),
    se_dv = se(overall_rating.c)
  ) %>% select(context, pill.e, n, mean_dv,sd_dv, se_dv)

rbind(stats_overall, rbind(stats_context, stats_pill))
```

```
## # A tibble: 5 x 6
##   context  pill.e     n   mean_dv     sd_dv      se_dv
## *   <chr>   <chr> <int>     <dbl>     <dbl>      <dbl>
## 1 overall overall   538 0.3976217 0.4851936 0.02091818
## 2      LT overall   538 0.3755005 0.5137469 0.02214920
## 3      ST overall   538 0.4198717 0.5229769 0.02254714
## 4 overall    -0.5   326 0.3508539 0.4837502 0.02679245
## 5 overall     0.5   212 0.4695382 0.4796947 0.03294557
```

Analyses H2: Pill

```
model_h2 <- lmer(rating.c ~ pill.e * context.e +
                      (1 | oc_id) +
                      (0 + context.e || oc_id) +
                      (1 | oc_id:block) +
                      (0 + context.e || oc_id:block) +
                      (1 | oc_id:block:date) +
                      (0 + context.e || oc_id:block:date),
                    data=filter(data_between, face_sex==theFaceSex, manip==th
eManip),
                    REML=FALSE)
summary_h2 <- summary(model_h2)
mySummary(summary_h2)
```

```
## [[1]]
## oc_id:block:date      oc_id:block            oc_id
##             3200              681              538
##
## [[2]]
##
##
## rowname               Estimate   Std. Error        df    t value    Pr(>|t|)  s
ig
## -----------------  ---------  -----------  --------  --------  ---------  -
---
## (Intercept)           0.407        0.021   538.264    19.221      0.000  *
**
## pill.e                0.116        0.042   538.264     2.747      0.006  *
*
## context.e             0.047        0.016   540.559     2.954      0.003  *
*
## pill.e:context.e     -0.027        0.032   540.559    -0.866      0.387
```

```
confint(model_h2, method = confint_method) %>% as.data.frame() %>% rownames_to
_column() %>% filter(!is.na(`2.5 %`))
```

```
##            rowname       2.5 %     97.5 %
## 1      (Intercept)  0.36507506 0.44798122
## 2           pill.e  0.03329955 0.19911186
## 3        context.e  0.01575287 0.07790052
## 4 pill.e:context.e -0.08959624 0.03469907
```

Note that the effect of OCP use (estimate = +0.116, p = .006) is in the opposite direction to what would be expected if fertility is positively associated with preference.

Analyses H2p: Pill (+ partnership status)

```
model_h2p <- lmer(rating.c ~ pill.e * context.e * partner.e +
                        (1 | oc_id) +
                        (0 + context.e || oc_id) +
                        (1 | oc_id:block) +
                        (0 + context.e || oc_id:block) +
                        (1 | oc_id:block:date) +
                        (0 + context.e || oc_id:block:date),
                     data=filter(data_between_partner, face_sex==theFaceSex, m
anip==theManip),
                     REML=FALSE)
summary_h2p <- summary(model_h2p)
mySummary(summary_h2p)
```

```
## [[1]]
## oc_id:block:date      oc_id:block           oc_id
##             2798              600             492
##
## [[2]]
##
##
## rowname                      Estimate   Std. Error        df   t value   P
r(>|t|)  sig
## ---------------------------  ---------  -----------  --------  --------  --
-------  ----
## (Intercept)                      0.436        0.023   491.341    18.884
0.000  ***
## pill.e                           0.119        0.046   491.341     2.583
0.010  *
## context.e                        0.049        0.017   483.540     2.813
0.005  **
## partner.e                        0.014        0.046   491.341     0.304
0.761
## pill.e:context.e                -0.019        0.035   483.540    -0.554
0.580
## pill.e:partner.e                -0.247        0.092   491.341    -2.672
0.008  **
## context.e:partner.e              0.068        0.035   483.540     1.952
0.051  +
## pill.e:context.e:partner.e       0.090        0.069   483.540     1.295
0.196
```

```
confint(model_h2p, method = confint_method) %>% as.data.frame() %>% rownames_t
o_column() %>% filter(!is.na(`2.5 %`))
```

```
##                      rowname         2.5 %      97.5 %
## 1                (Intercept)  0.3903766689  0.48079293
## 2                     pill.e  0.0287428868  0.20957542
## 3                  context.e  0.0147775065  0.08269070
## 4                  partner.e -0.0763792100  0.10445332
## 5           pill.e:context.e -0.0871204643  0.04870591
## 6           pill.e:partner.e -0.4273560163 -0.06569095
## 7        context.e:partner.e -0.0002664157  0.13555996
## 8 pill.e:context.e:partner.e -0.0460820309  0.22557073
```

Pill (single women only to interpret interaction)

```
model_h2p_single <- lmer(rating.c ~ pill.e * context.e +
                        (1 | oc_id) +
                        (0 + context.e || oc_id) +
                        (1 | oc_id:block) +
                        (0 + context.e || oc_id:block) +
                        (1 | oc_id:block:date) +
                        (0 + context.e || oc_id:block:date),
                     data=filter(data_between_partner, face_sex==theFaceSex,pa
rtner.e==-0.5, manip==theManip),
                     REML=FALSE,
                     # use nelder-mead optimziation (as in lmer v < 1)
                     control = lmerControl(optimizer ="Nelder_Mead")
                     )
summary_h2p_single <- summary(model_h2p_single)
mySummary(summary_h2p_single)
```

```
## [[1]]
## oc_id:block:date      oc_id:block            oc_id
##             1489              319              259
##
## [[2]]
##
##
## rowname              Estimate   Std. Error         df    t value   Pr(>|t|)  s
ig
## -----------------  ---------  -----------  --------  --------  ---------  -
---
## (Intercept)            0.428        0.032   251.741    13.304      0.000  *
**
## pill.e                 0.243        0.064   251.741     3.774      0.000  *
**
## context.e              0.016        0.022   252.842     0.743      0.458
## pill.e:context.e      -0.063        0.044   252.842    -1.439      0.151
```

```
confint(model_h2p_single, method = confint_method) %>% as.data.frame() %>% row
names_to_column() %>% filter(!is.na(`2.5 %`))
```

```
##            rowname       2.5 %     97.5 %
## 1      (Intercept)  0.36525917 0.49147636
## 2           pill.e  0.11678808 0.36922246
## 3        context.e -0.02663534 0.05917798
## 4 pill.e:context.e -0.14883821 0.02278844
```

Pill (partnered women only to interpret interaction)

```
model_h2p_partnered <- lmer(rating.c ~ pill.e * context.e +
                        (1 | oc_id) +
                        (0 + context.e || oc_id) +
                        (1 | oc_id:block) +
                        (0 + context.e || oc_id:block) +
                        (1 | oc_id:block:date) +
                        (0 + context.e || oc_id:block:date),
                    data=filter(data_between_partner,
                                face_sex==theFaceSex,
                                partner.e== 0.5,
                                manip==theManip),
                    REML=FALSE)
summary_h2p_partnered <- summary(model_h2p_partnered)
mySummary(summary_h2p_partnered)
```

```
## [[1]]
## oc_id:block:date      oc_id:block           oc_id
##             1309              281             233
##
## [[2]]
##
##
## rowname              Estimate   Std. Error        df    t value   Pr(>|t|)  s
ig
## -----------------  ---------  -----------  --------  --------  ---------  -
---
## (Intercept)            0.443        0.033   236.351    13.463      0.000  *
**
## pill.e                -0.004        0.066   236.351    -0.062      0.950
## context.e              0.082        0.027   231.207     3.066      0.002  *
*
## pill.e:context.e       0.026        0.053   231.207     0.493      0.622
```

```
confint(model_h2p_partnered, method = confint_method) %>% as.data.frame() %>%
rownames_to_column() %>% filter(!is.na(`2.5 %`))
```

```
##            rowname       2.5 %    97.5 %
## 1      (Intercept)  0.37822773 0.5071121
## 2           pill.e -0.13297648 0.1247922
## 3        context.e  0.02954214 0.1342130
## 4 pill.e:context.e -0.07832164 0.1310201
```

Hypothesis 3

Do preferences of women using the combined oral contraceptive pill change when they are taking inactive pills?

pill_break.e coding: using active pill= 0.5, using inactive pill/being on on a pill break = -0.5

Descriptive stats: data_pillbreak

```
# create mean DV for all ratings by oc_id
stats_overall <- filter(data_pillbreak, face_sex==theFaceSex, manip==theManip)
%>%
  group_by(oc_id) %>%
  summarise(
    overall_rating.c = mean(rating.c)
  ) %>%
  ungroup() %>%
  group_by() %>%
  summarise(
    context = "overall",
    n= n_distinct(oc_id),
    mean_dv = mean(overall_rating.c),
    sd_dv = sd(overall_rating.c),
    se_dv = se(overall_rating.c)
  )


# create mean DV splitting by context
stats_context <- filter(data_pillbreak, face_sex==theFaceSex, manip==theManip)
%>%
  group_by(oc_id, context) %>%
  summarise(
    context_rating.c = mean(rating.c)
  ) %>%
  group_by(context) %>%
  summarise(
    n= n_distinct(oc_id),
    mean_dv = mean(context_rating.c),
    sd_dv = sd(context_rating.c),
    se_dv = se(context_rating.c)
  )

rbind(stats_overall, stats_context)
```

```
## # A tibble: 3 x 5
##   context     n   mean_dv     sd_dv      se_dv
## *   <chr> <int>     <dbl>     <dbl>      <dbl>
## 1 overall   173 0.4380490 0.4488670 0.03412672
## 2      LT   173 0.4112107 0.4790548 0.03642186
## 3      ST   173 0.4650578 0.5110369 0.03885342
```

Analyses H3: Pill-break

```
model_h3 <- lmer(rating.c ~ context.e*pill_break.e +
                     (1 | oc_id) +
                     (0 + pill_break.e:context.e || oc_id) +
                     (1 | oc_id:block) +
                     (0 + pill_break.e:context.e || oc_id:block ) +
                     (1 | oc_id:block:date) +
                     (0 + context.e || oc_id:block:date ),
                 data=filter(data_pillbreak, face_sex==theFaceSex, manip==theM
anip),
                 REML=FALSE)
summary_h3 <- summary(model_h3)
mySummary(summary_h3)
```

```
## [[1]]
## oc_id:block:date      oc_id:block            oc_id
##             1019              206              173
##
## [[2]]
##
##
## rowname                    Estimate   Std. Error        df    t value   Pr(>|
t|)  sig
## -----------------------  ---------  -----------  --------  --------  ------
---  ----
## (Intercept)                  0.436        0.034   179.486    12.664      0.
000  ***
## context.e                    0.057        0.021   935.351     2.726      0.
007  **
## pill_break.e                 0.014        0.022   832.882     0.635      0.
526
## context.e:pill_break.e       0.007        0.047   208.006     0.149      0.
882
```

```
confint(model_h3, method = confint_method) %>% as.data.frame() %>% rownames_to
_column() %>% filter(!is.na(`2.5 %`))
```

```
##                  rowname       2.5 %     97.5 %
## 1            (Intercept)  0.36874879 0.50378236
## 2              context.e  0.01598821 0.09781643
## 3           pill_break.e -0.02932669 0.05743556
## 4 context.e:pill_break.e -0.08536960 0.09937692
```

Analyses H3p: Pill-break (+ partnership status)

```
model_h3p<-lmer(rating.c ~ context.e*pill_break.e * partner.e +
                                (1 | oc_id) +
                                (0 + pill_break.e*context.e || oc_id) +
                                (1 | oc_id:block) +
                                (0 + pill_break.e*context.e || oc_id:block ) +
                                (1 | oc_id:block:date) +
                                (0 + context.e || oc_id:block:date ),
                         data=filter(data_pillbreak_partner,
                                     face_sex==theFaceSex,
                                     manip==theManip),
                 REML=FALSE)
summary_h3p <- summary(model_h3p)
mySummary(summary_h3p)
```

```
## [[1]]
## oc_id:block:date      oc_id:block            oc_id
##              989              200              168
##
## [[2]]
##
##
## rowname                            Estimate   Std. Error        df   t val
ue   Pr(>|t|)  sig
## --------------------------------  ---------  -----------  --------  ------
--  ---------  ----
## (Intercept)                           0.431        0.035   180.905    12.2
98      0.000  ***
## context.e                             0.039        0.036   187.100     1.0
98      0.274
## pill_break.e                          0.019        0.024   147.433     0.8
05      0.422
## partner.e                             0.016        0.059   163.947     0.2
77      0.782
## context.e:pill_break.e                0.001        0.040   203.777     0.0
19      0.985
## context.e:partner.e                   0.145        0.070   205.922     2.0
87      0.038  *
## pill_break.e:partner.e               -0.023        0.048   184.083    -0.4
88      0.626
## context.e:pill_break.e:partner.e     -0.091        0.079   203.849    -1.1
47      0.253
```

```
confint(model_h3p, method = confint_method) %>% as.data.frame() %>% rownames_t
o_column() %>% filter(!is.na(`2.5 %`))
```

```
##                             rowname         2.5 %     97.5 %
## 1                       (Intercept)  0.362297291 0.49966784
## 2                         context.e -0.030972377 0.10982504
## 3                      pill_break.e -0.027716868 0.06631501
## 4                         partner.e -0.099015064 0.13157370
## 5            context.e:pill_break.e -0.076724321 0.07818687
## 6               context.e:partner.e  0.008806622 0.28148741
## 7            pill_break.e:partner.e -0.117070412 0.07035938
## 8 context.e:pill_break.e:partner.e -0.245577842 0.06429855
```

Pill break (single women only to interpret interaction)

```
model_h3p_single <- lmer(rating.c ~ context.e*pill_break.e  +
                               (1 | oc_id) +
                               (0 + pill_break.e*context.e || oc_id) +
                               (1 | oc_id:block) +
                               (0 + pill_break.e*context.e || oc_id:block ) +
                               (1 | oc_id:block:date) +
                               (0 + context.e || oc_id:block:date ),
                        data=filter(data_pillbreak_partner, partner.e=="-0.5"
,
                                     face_sex==theFaceSex, manip==theManip),
                 REML=FALSE)
summary_h3p_single <- summary(model_h3p_single)
mySummary(summary_h3p_single)
```

```
## [[1]]
## oc_id:block:date      oc_id:block            oc_id
##              335               68               61
##
## [[2]]
##
##
## rowname                     Estimate   Std. Error        df   t value   Pr(>|
t|)  sig
## ----------------------  ----------  -----------  --------  --------  ------
---  ----
## (Intercept)                  0.439        0.059    62.169     7.409      0.
000  ***
## context.e                   -0.038        0.039    67.475    -0.975      0.
333
## pill_break.e                 0.033        0.038   275.893     0.864      0.
388
## context.e:pill_break.e       0.053        0.060    58.735     0.876      0.
385
```

```
confint(model_h3p_single, method = confint_method) %>% as.data.frame() %>% row
names_to_column() %>% filter(!is.na(`2.5 %`))
```

```
##                  rowname       2.5 %     97.5 %
## 1            (Intercept)  0.32284883 0.55509562
## 2              context.e -0.11439962 0.03839673
## 3           pill_break.e -0.04168064 0.10746122
## 4 context.e:pill_break.e -0.06543218 0.17114999
```

Pill break (partnered women only to interpret interaction)

```
model_h3p_partnered <- lmer(rating.c ~ context.e*pill_break.e  +
                                (1 | oc_id) +
                                (0 + pill_break.e*context.e || oc_id) +
                                (1 | oc_id:block) +
                                (0 + pill_break.e*context.e || oc_id:block ) +
                                (1 | oc_id:block:date) +
                                (0 + context.e || oc_id:block:date ),
                         data=filter(data_pillbreak_partner,
                                     partner.e=="0.5",
                                     face_sex==theFaceSex,
                                     manip==theManip),
                     REML=FALSE,
                     # use nelder-mead optimziation (as in lmer v < 1)
                     control = lmerControl(optimizer ="Nelder_Mead"))
summary_h3p_partnered <- summary(model_h3p_partnered)
mySummary(summary_h3p_partnered)
```

```
## [[1]]
## oc_id:block:date      oc_id:block           oc_id
##              654              132             114
##
## [[2]]
##
##
## rowname                   Estimate   Std. Error        df   t value   Pr(>|
t|)  sig
## -----------------------  ---------  -----------  --------  --------  ------
---  ----
## (Intercept)                  0.413        0.040   119.511    10.338      0.
000  ***
## context.e                    0.109        0.047   121.140     2.290      0.
024  *
## pill_break.e                 0.004        0.030    99.365     0.118      0.
906
## context.e:pill_break.e      -0.049        0.047   130.407    -1.031      0.
304
```

```
confint(model_h3p_partnered, method = confint_method) %>% as.data.frame() %>%
rownames_to_column() %>% filter(!is.na(`2.5 %`))
```

```
##                   rowname       2.5 %     97.5 %
## 1            (Intercept)  0.33505338 0.49180959
## 2              context.e  0.01564686 0.20152335
## 3           pill_break.e -0.05493096 0.06198526
## 4 context.e:pill_break.e -0.14125492 0.04385052
```

Hypothesis 4

Do preferences change when women start or stop using the combined oral contraceptive pill?

Descriptive stats: data_pill_switchers

```
# create mean DV for all ratings by oc_id
stats_overall <- filter(data_pill_switchers, face_sex==theFaceSex, manip==theM
anip) %>%
  group_by(oc_id) %>%
  summarise(
    overall_rating.c = mean(rating.c)
  ) %>%
  ungroup() %>%
  group_by() %>%
  summarise(
    context = "overall",
    n= n_distinct(oc_id),
    mean_dv = mean(overall_rating.c),
    sd_dv = sd(overall_rating.c),
    se_dv = se(overall_rating.c)
  )


# create mean DV splitting by context
stats_context <- filter(data_pill_switchers, face_sex==theFaceSex, manip==theM
anip) %>%
  group_by(oc_id, context) %>%
  summarise(
    context_rating.c = mean(rating.c)
  ) %>%
  group_by(context) %>%
  summarise(
    n= n_distinct(oc_id),
    mean_dv = mean(context_rating.c),
    sd_dv = sd(context_rating.c),
    se_dv = se(context_rating.c)
  )

rbind(stats_overall, stats_context)
```

```
## # A tibble: 3 x 5
##   context     n   mean_dv     sd_dv      se_dv
## *   <chr> <int>     <dbl>     <dbl>      <dbl>
## 1 overall    45 0.3643366 0.4321600 0.06442261
## 2      LT    45 0.3365961 0.4811474 0.07172522
## 3      ST    45 0.3917319 0.4190430 0.06246725
```

Interval between pill use and non-use testing blocks

```
switchers_date_diffs <- filter(data_pill_switchers, face_sex==theFaceSex, mani
p==theManip) %>%
  group_by(oc_id, block_pill, direction.e) %>%
  summarise(
    min_date = min(date),
    max_date = max(date),
    the_date = ifelse(
        mean(direction.e) == -.5,
        ifelse(block_pill == 0, max_date, min_date),
        ifelse(block_pill == 1, max_date, min_date)
      )
  ) %>%
  ungroup() %>%
  mutate(block_pill = paste0("hc", block_pill)) %>%
  select(oc_id, block_pill, the_date) %>%
  spread(block_pill, the_date) %>%
  mutate( date_diff = abs(interval(ymd(hc0), ymd(hc1)) / ddays(1)))

switchers_date_diffs %>%
  group_by() %>%
  summarise(
    mean = mean(date_diff),
    sd = sd(date_diff),
    se = se(date_diff),
    min = min(date_diff),
    max = max(date_diff)
  ) %>% gather("date_diff", "value", 1:length(.)) %>%
    mutate(value = round(value, 4))
```

```
## # A tibble: 5 x 2
##   date_diff     value
##       <chr>     <dbl>
## 1      mean  360.3111
## 2        sd  282.0838
## 3        se   42.0506
## 4       min   56.0000
## 5       max 1113.0000
```

Analyses H4: Pill-switch

Within-subject change in pill use: (not considering partnership status)

```
model_h4 <- lmer(rating.c ~ pill.e*context.e + direction.e +
                          (1 | oc_id) +
                          (0 + context.e:pill.e || oc_id) +
                          (1 | oc_id:block) +
                          (0 + context.e || oc_id:block) +
                          (1 | oc_id:block:date) +
                          (0 + context.e || oc_id:block:date),
                     data=filter(data_pill_switchers, face_sex==theFaceSex, ma
nip==theManip),
                     REML=FALSE)
summary_h4 <- summary(model_h4)
mySummary(summary_h4)
```

```
## [[1]]
## oc_id:block:date      oc_id:block            oc_id
##              529              107               45
##
## [[2]]
##
##
## rowname              Estimate   Std. Error        df    t value    Pr(>|t|)  s
ig
## -----------------  ---------  -----------  --------  --------  ---------  -
---
## (Intercept)            0.389        0.067    45.605     5.826      0.000  *
**
## pill.e                 0.077        0.049    63.381     1.570      0.121
## context.e              0.056        0.031   107.290     1.813      0.073  +
## direction.e            0.147        0.134    45.848     1.102      0.276
## pill.e:context.e       0.087        0.061   107.290     1.424      0.157
```

```
confint(model_h4, method = confint_method) %>% as.data.frame() %>% rownames_to
_column() %>% filter(!is.na(`2.5 %`))
```

```
##            rowname        2.5 %    97.5 %
## 1      (Intercept)  0.258416339 0.5204390
## 2           pill.e -0.019123218 0.1732065
## 3        context.e -0.004497919 0.1155415
## 4      direction.e -0.114877104 0.4098350
## 5 pill.e:context.e -0.032826771 0.2072520
```

Analyses H4p: Pill-switch (+ partnership status change)

Within-subject change in pill use: (considering possible effects of change in partnership status)

```
model_h4p <- lmer(rating.c ~ pill.e * context.e + partner.e + direction.e +
                          (1 | oc_id) +
                          (0 + context.e:pill.e + partner.e || oc_id) +
                          (1 | oc_id:block) +
                          (0 + context.e || oc_id:block) +
                          (1 | oc_id:block:date) +
                          (0 + context.e || oc_id:block:date),
                     data=filter(data_pill_switchers_partner,
                                 face_sex==theFaceSex,
                                 manip==theManip),
                     REML=FALSE)
summary_h4p <- summary(model_h4p)
mySummary(summary_h4p)
```

```
## [[1]]
## oc_id:block:date      oc_id:block            oc_id
##              399               81               35
##
## [[2]]
##
##
## rowname              Estimate   Std. Error       df   t value   Pr(>|t|)  si
g
## -----------------  ---------  -----------  -------  --------  ---------  --
--
## (Intercept)            0.453        0.078   35.645     5.822      0.000  **
*
## pill.e                 0.005        0.063   49.679     0.076      0.940
## context.e              0.027        0.027   81.046     0.975      0.332
## partner.e              0.123        0.088   35.706     1.392      0.172
## direction.e            0.179        0.156   35.712     1.149      0.258
## pill.e:context.e       0.021        0.055   81.046     0.389      0.698
```

```
confint(model_h4p, method = confint_method) %>% as.data.frame() %>% rownames_t
o_column() %>% filter(!is.na(`2.5 %`))
```

```
##            rowname       2.5 %     97.5 %
## 1      (Intercept)  0.30043773 0.60539761
## 2           pill.e -0.11920153 0.12879195
## 3        context.e -0.02685847 0.08002384
## 4        partner.e -0.04994438 0.29496199
## 5      direction.e -0.12624575 0.48381275
## 6 pill.e:context.e -0.08568008 0.12808420
```
